# Hérnia de disco lombar: O que há de novo no tratamento cirúrgico

**DOI:** 10.1055/s-0046-1824522

**Published:** 2026-10-06

**Authors:** Fábio Antônio Vieira, Breno Augusto Giese Ribeiro, Renato Hiroshi Salvioni Ueta, Eduardo Barros Puertas

**Affiliations:** 1 Departamento de Ortopedia e Traumatologia, Escola Paulista de Medicina, Universidade Federal de São Paulo, São Paulo, SP, Brasil

**Keywords:** ciática, discectomia, hérnia de disco, diskectomy, herniated disc, sciatica

## Abstract

A hérnia de disco lombar é uma das principais causas de dor radicular e incapacidade na população adulta, com impacto expressivo no sistema de saúde e na produtividade laboral. O manejo inicial é predominantemente conservador, mas uma parcela dos pacientes pode evoluir com sintomas refratários ou déficit neurológico progressivo, necessitando de intervenção cirúrgica. Ao longo das últimas décadas, técnicas minimamente invasivas, como a cirurgia endoscópica, revolucionaram o tratamento com menor agressão tecidual, recuperação acelerada e menores taxas de complicações. Este artigo revisa a evolução das técnicas cirúrgicas, destacando evidências comparativas entre elas, bem como o papel emergente de novas tecnologias.

## Abstract (en)

Lumbar disc herniation is one of the main causes of root pain and disability in the adult population, with a significant impact on the healthcare system and labor productivity. Initial management is predominantly conservative, but a portion of patients may evolve with refractory symptoms or progressive neurological deficit, requiring surgical intervention. Over the past few decades, minimally invasive techniques, such as endoscopic surgery, have revolutionized treatment with less tissue damage, accelerated recovery, and lower complication rates. This article reviews the evolution of surgical techniques, highlighting comparative evidence among them and the emerging role of new technologies.

## Introdução

A hérnia de disco lombar apresenta uma alta incidência na população, sendo a principal causa de dor lombar e radiculopatia entre adultos de 30 a 50 anos. A fisiopatologia da hérnia é complexa e envolve alterações biomecânicas, bioquímicas e estruturais progressivas. 1 O processo degenerativo do disco intervertebral (DIV) leva a desidratação do núcleo pulposo (NP), com diminuição da altura do DIV e a sobrecarga do ânulo fibroso (AF), levando a rotura de suas fibras. 2 3 As fissuras permitem a extrusão do conteúdo do NP para o canal vertebral, o que pode provocar compressão mecânica da raiz nervosa. 3 Os mediadores inflamatórios liberados durante a degeneração discal exercem papel fundamental, podendo gerar dor de padrão radicular mesmo em casos sem compressão exuberante. 4

Clinicamente o paciente apresenta dor lombar irradiada seguindo um dermátomo específico, podendo evoluir com déficit sensitivo ou motor. 3 5 Nos casos de hérnias volumosas ou sequestros discais, o quadro pode evoluir para síndrome da cauda equina, constituindo emergência devido ao risco de sequelas permanentes. 6 Os níveis L4 a L5 e L5 a S1 são responsáveis por cerca de 90% das hérnias discais lombares, o que se relaciona à maior mobilidade e estresse biomecânico desses segmentos. 2

A história natural da hérnia de disco lombar é, em geral, favorável. Cerca de 80% dos pacientes apresentam melhora espontânea em 3 meses e até 90% em 1 ano. 3 A reabsorção espontânea do fragmento pode ocorrer, especialmente em extrusões migradas ou sequestradas, nas quais o efeito imunológico sobre o material exposto acelera a absorção. 7 O tratamento conservador é multimodal, composto por analgésicos, anti-inflamatórios, fisioterapia e atividade física, devendo ser a abordagem inicial padrão. 8 9 No entanto, aproximadamente 20% dos pacientes evoluem com sintomas persistentes após 12 semanas de tratamento ou apresentam déficit neurológico progressivo, tornando necessária a intervenção cirúrgica. 3 10 11

Historicamente, a discectomia aberta é a forma clássica de abordagem, apresentando altas taxas de melhora clínica e resultados satisfatórios. 12 A microdiscectomia representou grande avanço técnico ao introduzir o microscópio para menor agressão muscular e melhor visualização cirúrgica. 13 A partir dos anos 2000, técnicas minimamente invasivas, incluindo microdiscectomia tubular e discectomia endoscópica ( Fig. 1 ), consolidaram-se como alternativas eficazes, permitindo menor lesão tecidual, menor sangramento e recuperação mais rápida. 14 15 16

**Fig. 1:**
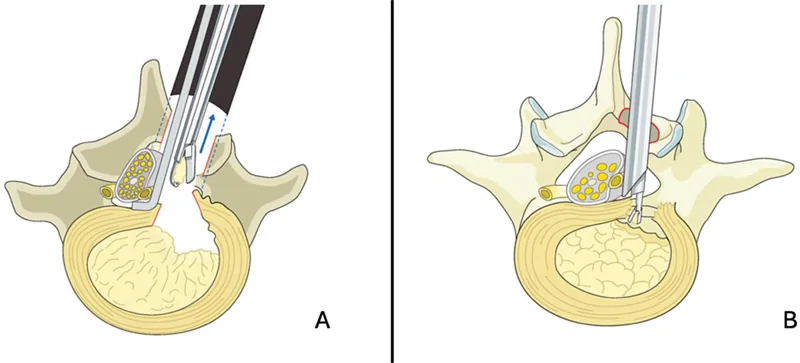
Representação da discectomia de hérnia centro-lateral. ( **A** ) Microdiscectomia tubular. ( **B** ) Discetomia endoscópica. **Fonte** : Balsano M et al. Disponível em https://surgeryreference.aofoundation.org/spine/degenerative/lumbar/disc-herniation-central-posterolateral/ .

Outros avanços incluem o desenvolvimento de dispositivos de fechamento anular, voltados à prevenção de recidivas em pacientes com grandes defeitos no ânulo, 17 18 e o crescente interesse em terapias de medicina regenerativa, que exploram o potencial de células sinalizadoras para restaurar a biologia do núcleo pulposo e modular a inflamação. 19 Em casos de hérnias de disco com instabilidade segmentar ou degeneração avançada do DIV, as artrodeses seguem desempenhando papel essencial. 20

Assim, o cenário atual do tratamento cirúrgico da hérnia lombar é marcado por um rápido desenvolvimento tecnológico e pela aproximação entre abordagens mecânicas e biológicas, ampliando o arsenal terapêutico e permitindo estratégias cada vez mais individualizadas.

## Discectomia

### Microdiscectomia tubular

A microdiscectomia tubular emprega dilatadores sequenciais para afastamento sem trauma da musculatura paravertebral. Após posicionamento da cânula de trabalho e visualização microscópica, realiza-se laminotomia mínima, flavectomia e remoção do fragmento herniado ( Fig. 2 ). 21

**Fig. 2:**
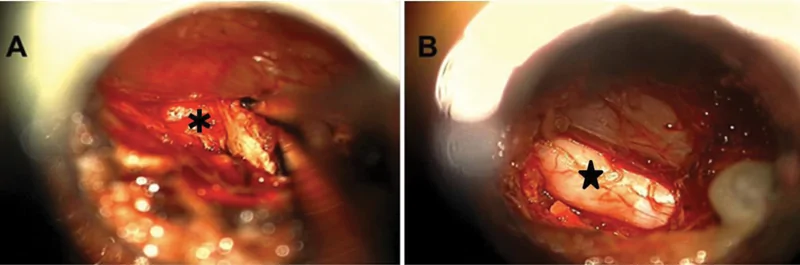
Imagens intraoperatórias de microdiscectomia tubular. ( **A** ) Identificação do fragmento herniado. ( **B** ) Nervo livre após a discectomia. ★Raiz lombar. ✱Hérnia de disco.

As principais limitações da técnica tubular incluem campo cirúrgico mais restrito que dificulta ampliação da exposição, caso necessário, e hérnias laterais ou em múltiplos níveis para casos de fragmentos migrados. 22 A presença do afastador tubular pode se apresentar como dificuldade para manipulação e exploração dos níveis, especialmente para cirurgiões não familiarizados com o procedimento. Assim, a técnica tubular apresenta curva de aprendizado mais longa que a discectomia aberta. 22

Apesar de menor agressão tecidual, os resultados clínicos são semelhantes aos da microdiscectomia tradicional, sem superioridade da técnica tubular. Estudos comparativos não identificam diferenças significativas quanto aos desfechos de tempo cirúrgico, sangramento, resultados funcionais e taxa de recorrência. 21 23 Outros estudos demonstram melhores resultados na dor pós-operatória, com base na escala visual analógica (EVA) para dor lombar e dor irradiada na microdiscectomia convencional em comparação à tubular. 22

A discectomia tubular é, portanto, alternativa segura e eficaz para o tratamento da hérnia de disco lombar. Apresentando resultados comparáveis, porém com curva de aprendizado mais longa em relação à microdiscectomia convencional.

### Cirurgia endoscópica

A cirurgia endoscópica representa o avanço mais significativo desde a microdiscectomia. As primeiras descrições da técnica datam da década de 1980 e 1990, porém foi a partir dos anos 2000 com a evolução tecnológica dos instrumentais que ocorreram os principais avanços. 24 25 As abordagens podem ser realizadas por portal único, como a discectomia totalmente endoscópica (FED, do inglês *full-endoscopic* *discectom* y) ou técnica biportal ((UBE, do inglês *unilateral biportal endoscopy* ), com incisões de 6 a 10 mm, e mínima agressão local. 24

Dois acessos clássicos foram descritos: transforaminal e interlaminar, escolhidos conforme nível e morfologia da hérnia. 26 A via transforaminal possibilita acesso direto ao disco pelo triângulo de Kambin, sendo o acesso de escolha para hérnias foraminais e extraforaminais e níveis lombares altos onde a área do forame é maior. A via interlaminar favorece o tratamento de hérnias centrais e centro-laterais, sobretudo nos níveis lombares baixos onde a janela interlaminar é maior ( Fig. 3 ). 25 26

**Fig. 3:**
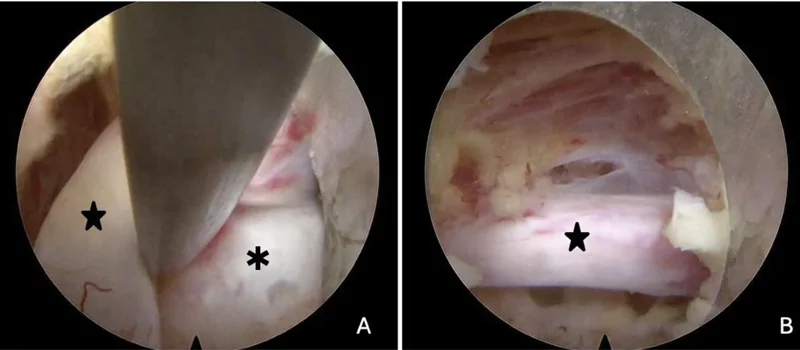
Imagem intraoperatória de endoscopia interlaminar lombar. ( **A** ) Afastamento da raiz e exposição da hernia de disco. ( **B** ) Nervo livre após discectomia. ★Raiz lombar. ✱Hérnia de disco.

Os resultados clínicos da cirurgia endoscópica comparados a microdiscectomia mostram resultados semelhantes quando se analisam a dor no pós-operatório (EVA) e escores funcionais, como o índice de Owestry (ODI, do inglês Owestry Disability Index), no médio e longo prazo melhores para os pacientes submetidos a endoscopia. 14 15 16 Outros fatores positivos a favor da endoscopia são o menor tempo de permanência hospitalar e de retorno às atividades laborais, menor sangramento intraoperatório e menor taxa de complicações. 14 15 27 Com relação à recorrência, definida como retorno dos sintomas no mesmo nível operado após período livre dos sintomas, as taxas são semelhantes entre as técnicas, de 3 a 5% no período de 5 anos. 12 16 27 28

A cirurgia endoscópica requer instrumentais de maior tecnologia que representam procedimento de maior custo inicial do que outras opções de discectomia. 29 30 Porém, o retorno precoce ao trabalho, aliado ao menor período de internação hospitalar fazem com que os custos totais do procedimento, que incluem o custo social e perda de produtividade, tendem a ser menores nas cirurgias endoscópicas. 29 31 Análises de custo-efetividade demonstram que a cirurgia endoscópica é mais eficaz e menos custosa em 12 meses do que a microdiscectomia. 30 31

A cirurgia endoscópica apresenta maior curva de aprendizado em comparação com outras técnicas. Em média, são considerados cerca de 25 casos para domínio da técnica biportal e 40 para a FED, o que pode aumentar a taxa de complicações e tempo cirúrgico nos casos iniciais. 32 Já para a microdiscectomia são necessários de 10 a 18 casos, facilitando sua adesão e reduzindo variabilidade de resultados. 33

## Medicina regenerativa

O uso da medicina regenerativa para o tratamento de patologias da coluna ainda segue em investigação, com estudos voltados para a aplicação de células troncos mesenquimais (CTMs), concentrados de medula óssea (CMOs) e plasma rico em plaquetas (PRP). Diversos estudos buscam avaliar os resultados do controle de dor lombar em pacientes com doença degenerativa do disco, com resultados ainda insuficientes para validar seu uso rotineiro. 19

Para o tratamento de pacientes com queixa de radiculopatia por hérnia de disco lombar há evidências que apontam em melhor controle da dor radicular (EVA) após infiltração transforaminal com PRP quando comparado ao uso de corticoide, porém sem alteração de função (ODI) ou no controle da dor lombar. 34

Outra forma de empregar a medicina regenerativa avaliada em alguns estudos é a utilização do PRP após procedimentos endoscópicos de discectomia. Um dos poucos estudos clínicos randomizados mostrou que não houve diferença nos escores clínicos (ODI e EVA) entre os pacientes que foram submetidos ao PRP comparados aos que não foram. Único achado relevante deste estudo foi uma melhora na escala de Pfirrmann com base nas imagens de ressonância magnética do grupo que utilizou PRP. 35

Apesar de resultados promissores em outras áreas, a eficácia da medicina regenerativa para tratamento da hérnia de disco lombar ainda carece de resultados e estudos robustos, com padronização dos protocolos de aplicação, dosagem e frequência.

## Dispositivos de fechamento anular

Dispositivos e tecnologias para reparo do defeito anular foram desenvolvidos com o objetivo de cobrir a falha no ânulo fibroso e evitar a recidiva. Dentre os sistemas disponíveis destacam-se a sutura do ânulo e dispositivos de fechamento. As principais indicações são para defeitos acima de 6mm com altura discal preservada, fatores associados a aumento da recidiva da hérnia da disco. 18

A sutura do ânulo pode ser realizada por via endoscópica ou microcirúrgica. Diversas técnicas de sutura são descritas, com ancoragem óssea, fixação apenas no anulo ou sutura conjunta do ligamento longitudinal posterior (LLP). 36 Estudos iniciais demonstram segurança da técnica, porém com resultados insuficientes para corroborar seu uso como rotina na prática cirúrgica. 37

Os dispositivos projetados para fechar o defeito anular após discectomia são implantes de ancoragem no corpo vertebral que preenchem a falha no ânulo. Ensaios clínicos demonstram redução de recidiva para 7 a 12% e queda nas taxas de reoperação de 13 a 16% para 5 a 6%. 18 A taxa de complicações graves não aumentam, e a função clínica permanece semelhante entre pacientes com e sem o dispositivo. 18 Tais dispositivos contam com aprovação da Food and Drug Adminstration (FDA) para uso nos EUA, porém ainda não estão disponíveis no mercado brasileiro.

## Artrodese lombar

Diretrizes norte-americanas não recomendam artrodese para radiculopatia por hérnia discal, sendo indicada para instabilidade radiográfica, recidivas múltiplas ou descompressão óssea ampla. 38 A artrodese reduz recidivas e instabilidade pós-operatória quando comparada à discectomia isolada, sobretudo após recorrências múltiplas (duas ou mais), mas não melhora os desfechos funcionais, já que o alívio restringe-se principalmente à dor lombar, sem impacto significativo na dor radicular ou nos índices de funcionalidade. 20 A artrodese resulta em maior tempo cirúrgico, maior perda sanguínea, internação prolongada e risco aumentado de complicações, como infecção, mal posicionamento dos implantes e degeneração do nível adjacente. 39

Seu benefício funcional é modesto e deve ser considerado devido ao maior risco de complicações. Em trabalhadores braçais, a artrodese pode aliviar a dor lombar, porém resulta em menor taxa de retorno ao trabalho, maior afastamento, maior uso de medicação analgésica e custos mais elevados, enquanto a discectomia oferece recuperação mais rápida, apesar do maior risco de nova recorrência em atividades fisicamente exigentes. 37

## Considerações finais

O tratamento cirúrgico da hérnia de disco lombar está em constante evolução, com o desenvolvimento de técnicas cada vez mais avançadas. A microdiscectomia continua como técnica segura, eficaz, amplamente difundida e disponível. A cirurgia endoscópica cada vez mais se dissemina entre os cirurgiões e apresenta resultados superiores na recuperação a curto prazo, com retorno precoce do paciente às suas funções, associado a maior custo-efetividade no período de 12 meses, um aspecto importante a ser considerado no cenário de saúde.

As técnicas de fechamento de defeito no ânulo seguem em estudo, assim como a medicina regenerativa, e são necessários estudos de qualidade para avaliar sua real eficácia. A artrodese segue como tratamento de exceção para hérnia de disco, sendo indicada para casos selecionados.

Por fim, a escolha da técnica cirúrgica deve ser definida a partir da experiência de cada cirurgião e individualizada para cada paciente, considerando o quadro clínico, fatores de risco associados, correlação clínico-radiológica e resultados esperados, alinhados com a expectativa do paciente.
